# The complete chloroplast genome of a distylous-homostylous species, *Primula homogama* (Primulaceae)

**DOI:** 10.1080/23802359.2020.1869614

**Published:** 2021-02-08

**Authors:** Hua-Ying Sun, Li Zhong, Yong-Jie Guo, Wei Zhou, Zhi-Kun Wu

**Affiliations:** a School of Chinese Materia Medica, Yunnan University of Chinese Medicine, Yunnan, China; b Kunming Institute of Botany, Chinese Academy of Sciences, Yunnan, China; c University of Chinese Academy of Sciences, Beijing, China; d Department of Pharmacy, Guizhou University of Traditional Chinese Medicine, Guizhou, China

**Keywords:** Complete chloroplast genome, phylogenetic analysis, *Primula homogama*

## Abstract

*Primula homogama* F. H. Chen & C. M. Hu (Primulaceae) is endemic to the Emei Mountain of China. In this study, we characterized the complete chloroplast genome of *P. homogama* based on next-generation sequencing (NGS). The complete chloroplast genome of *P. homogama* was 154,677 bp in size with a typical quadripartite structure, containing a large single-copy (LSC) region of 85,299 bp and a small single-copy (SSC) region of 17,816 bp. These two regions were separated by a pair of inverted repeat regions (IRs), each of 25,781 bp. A total of 130 functional genes were encoded, consisted of 86 protein-coding genes (PCG), 36 tRNA genes, and eight ribosomal RNA (rRNA) genes.

*Primula homogama* F. H. Chen & C. M. Hu, belonging to sect. *Souliei* of Primulaceae, is a perennial herb species endemic to the Emei Mountain of China (Hu and Kelso 1996). Contrary to the distyly conditions for vast majority (92%) of *Primula* species (∼400–500), *P. homogama* was formerly recognized as monomorphic for stylar condition and homostyly with populations comprising a single floral form with anthers and stigmas close together within a single flower. These plants are generally self-compatible and predominantly selfing as a result of autonomous self-pollination (Ganders 1979). However, our preliminary observations of *P. homogama* has revealed the mixed populations containing both distylous and homostylous forms. Thus, the species represent a recent transition from distyly to homostyly (Zhong et al. 2019). The complete plastome could provide valuable genomic information for the phylogeny and population structure of this distylous-homostylous species.

Fresh leaves tissue of *P. homogama* were collected from a wild population (103°15′00″E, 29°30′00″N) in Hongya County, Meishan City, Sichuan Province. We isolated total genomic DNA following a modified CTAB protocol (Doyle 1991). The purified genomic DNA was sheared into *c*. 500 bp fragments to construct a paired-end (PE) library according to the Nextera XT sample preparation procedures (Illumina, San Diego, CA). We generated the PE reads of 150 bp using HiSeq X-Ten sequencer (Illumina, San Diego, California, USA).

In all, 3.53 Gb of raw sequence data were obtained. Reads were assembled into contigs using program CLC Genomics Workbench version 8.5.1 (CLC Inc, Arhus, Denmark). We annotated the complete plastome using the DOGMA pipeline (Wyman et al. 2004) and validated by comparing with the chloroplast genome of *P. sinensis* Sabine ex Lindl. (KU321892) (Liu et al. 2016). We determined transfer RNAs using tRNAscan-SE (Schattner et al. 2005). The annotated chloroplast genome of *P. homogama* was deposited into GenBank with the accession number MW167117.

The complete chloroplast genome of *P. homogama* was 154,677 bp in size with a typical quadripartite structure, containing a large single-copy (LSC) region of 85,299 bp and a small single-copy (SSC) region of 17816 bp. These two regions were separated by a pair of inverted repeat regions (IRs), each of 25,781 bp. A total of 130 functional genes were encoded, consisted of 86 protein-coding genes (PCG), 36 tRNA genes, and eight ribosomal RNA (rRNA) genes. Of these genes, 18 genes were duplicated in the IR region, including seven protein-coding genes, seven tRNA genes, and four rRNA genes. Eighteen genes contain one or two introns. The overall GC content of the chloroplast genome was 36.9%, whereas the corresponding values of the LSC, SSC, and IR regions were 34.8, 30.6, and 42.7%, respectively.

The maximum likelihood (ML) phylogenetic tree was reconstructed by RAxML (Stamatakis 2014) based on 23 complete chloroplast genomes of *Primula* species and *Androsace laxa* as outgroup. The phylogenetic results indicated that the sampled *Primula* species clustered as a monophyletic clade with high support (100%), and *P. homogama* formed a clade (100% bootstrap value) with *P. knuthiana*, *P. pulchella*, *P. veris* and *P. denticulata* subsp. *sinodenticulata* (Figure 1). This published *P. homogama* chloroplast genome will provide useful information for phylogenetic and evolutionary studies in *Primula*.

**Figure 1.**
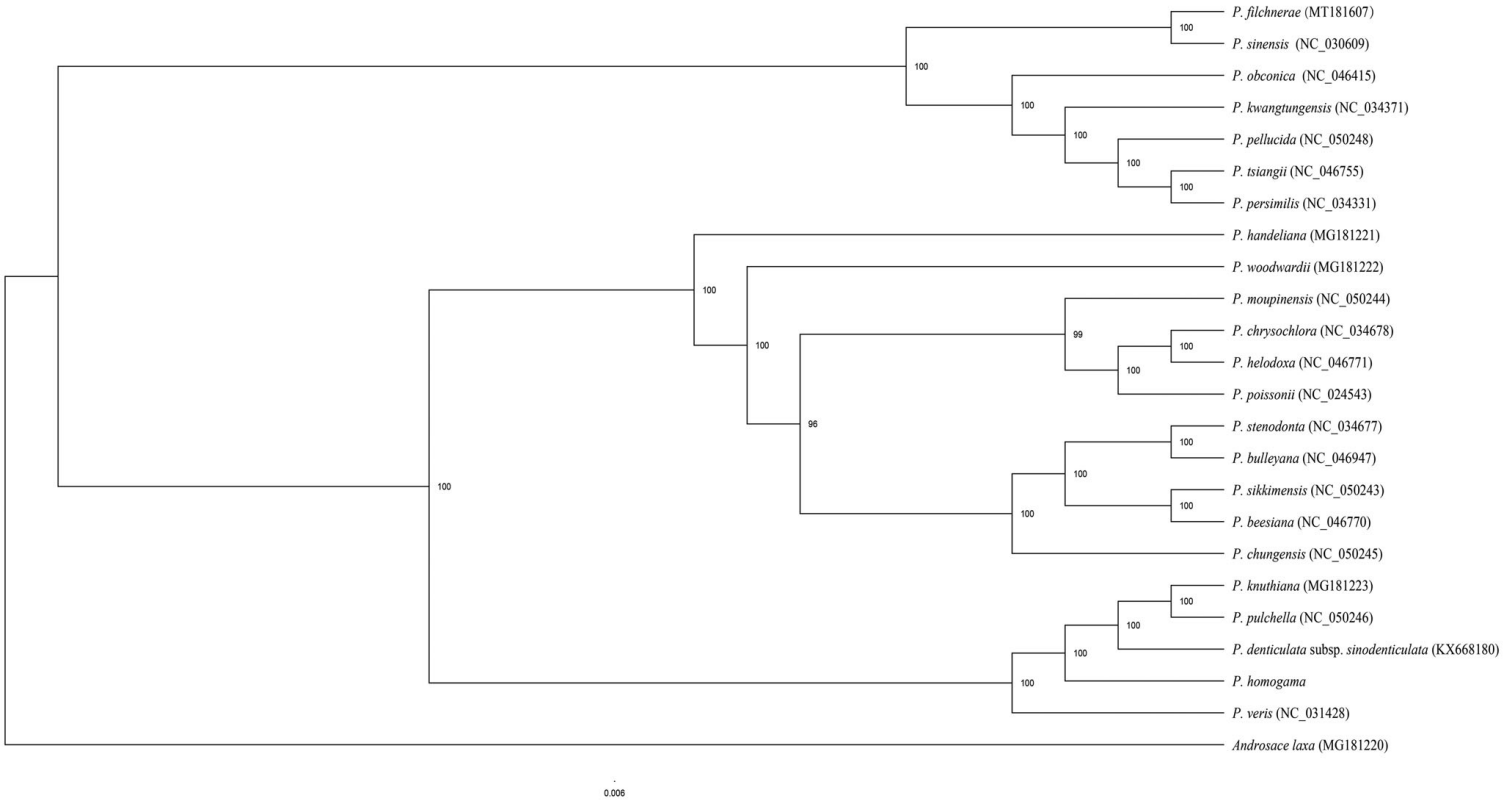
The maximum likelihood (ML) tree inferred from 24 complete chloroplast genome with 1000 bootstrap replicates. The number on each node indicates the bootstrap value.

## Data Availability

The genome sequence data that support the findings of this study are openly available in GenBank of NCBI at (https://www.ncbi.nlm.nih.gov/) under the accession no. MW167117. The associated **BioProject**, **SRA**, and **Bio-Sample** numbers are SRX9518288, PRJNA678458, and SAMN16801853 respectively.
